# Supplementary material for: Presence and abundance of bacteria with the Type VI secretion system in a coastal environment and in the global oceans
Source: PLoS One. 2020 Dec 22;15(12):e0244217. doi: 10.1371/journal.pone.0244217 (PMC7755280; doi:10.1371/journal.pone.0244217)
Supplement: S3 Table — (DOCX) [file pone.0244217.s003.docx]

**S3 Table.** Environmental parameter ranges, means, and standard deviations for the time period from 8 January, 2014 through 16 December, 2015.

|  |  |  |  |  |
| --- | --- | --- | --- | --- |
| Name | Unit | Abbreviation | Mean (± Standard Deviation) | Range |
| Nutrients |  |  |  |  |
| Ammonia | µM | NH3 | 3.53 (±1.97) | 0.34-9.57 |
| Nitrate | µM | NO3 | 2.89 (±3.49) | 0.02-22.5 |
| Phosphate | µM | PO4 | 0.57 (±0.41) | 0.01-1.76 |
| Silicate | µM | Si | 9.83 (±5.86) | 1.2-38.56 |
| Water Temperature | ˚C | Temp. | 14.6 (±2.3) | 9.7-18.7 |
| Productivity |  |  |  |  |
| Toxic Pseudo-nitzschia | cells/L | Toxic_PN | 27829 (±79386) | 0-491450 |
| Chlorophyll | mg/m^3^ | chl | 4.77 (±5.27) | 0.81-48.89 |
|  |  |  |  |  |

Data publicly available from: https://erddap.sccoos.org/erddap/tabledap/HABs-SantaCruzWharf.html
