# Supplementary material for: Presence and abundance of bacteria with the Type VI secretion system in a coastal environment and in the global oceans
Source: PLoS One. 2020 Dec 22;15(12):e0244217. doi: 10.1371/journal.pone.0244217 (PMC7755280; doi:10.1371/journal.pone.0244217)
Supplement: Attachment — Submitted filename: Response to reviewers.docx [file pone.0244217.s005.docx]

***Journal Requirements:***

*When submitting your revision, we need you to address these additional requirements.*

*1. Please ensure that your manuscript meets PLOS ONE's style requirements, including those for file naming. The PLOS ONE style templates can be found at*

[*https://journals.plos.org/plosone/s/file?id=wjVg/PLOSOne_formatting_sample_main_body.pdf*](https://journals.plos.org/plosone/s/file?id=wjVg/PLOSOne_formatting_sample_main_body.pdf) *and*

[*https://journals.plos.org/plosone/s/file?id=ba62/PLOSOne_formatting_sample_title_authors_affiliations.pdf*](https://journals.plos.org/plosone/s/file?id=ba62/PLOSOne_formatting_sample_title_authors_affiliations.pdf)

 - we have read PLOS One requirements and adhered thoroughly to the instructions, especially for file naming.

*2. In your Methods section, please provide additional location information of the collection site, including geographic coordinates for the data set if available.*

*-* Coordinates have been added to the sampling site location information on Line 104

*3. In your Methods section, please provide additional information regarding the permits you obtained for the work. Please ensure you have included the full name of the authority that approved the collection site access and, if no permits were required, a brief statement explaining why.*

*-*No permits were required for gathering samples, as Santa Cruz Wharf is open to the public. A statement to this affect has been added to the methods section in Line 105

*4. Please ensure that you refer to Figure 3 in your text as, if accepted, production will need this reference to link the reader to the figure.*

*-* Previously, Figure 3 erroneously called it figure 4 in the text. This has been corrected to Fig 3. See line 327.

*5. Please include a caption for figure 3.*

- We have provided a caption for corrected fig 3 (see above). See Line 328.

***Comments to the Author***

*Reviewer #1: In this study, the authors use bacterial DNA isolated from marine water samples from several locations and at different time points to analyze the relative abundance of genera carrying genes encoding structural components of the type VI secretion system (T6SS). One of their main findings is that T6SS-carrying bacteria appear to be more prevalent in the particle-attached fraction of their samples. They also correlate relative abundances with several environmental parameters, such as nutrient concentrations, temperature etc.

The study investigates the potential influence of an interesting bacterial phenotype (the T6SS machinery) on bacterial communities in natural environments, and I find the results to be intriguing and the manuscript well-written overall.

My only major complaints would be the following:

a) Relative abundances generally, and putative predation networks specifically. Fig. 6 shows interaction analyses based on relative abundance data of T6SS-containing bacteria in different samples and different time points (Santa Cruz wharf dataset). I am a bit sceptical of this analysis and the claims based on it, as these putative interaction networks are purely based on high-throughput sequencing data, and the relationship between absolute abundance in the environment (i.e. what we are actually interested in) and the relative abundance after sequencing (what we analyse) is often not predictable. See e.g. Fig. 1B and 1C here:* [*https://www.frontiersin.org/articles/10.3389/fmicb.2017.02224/full*](https://www.frontiersin.org/articles/10.3389/fmicb.2017.02224/full)

- We do agree that HTS 16S-sequences are compositional data and do not reflect the exact number of molecules of bacteria in a particular sample. Hence relative abundances have inherent limitations and may not be suitable dataset to studying correlations. We followed the advice of Gloor et al. 2017 paper (as pointed out by the reviewer) and have revised the analysis using centered log-ratio transformations which uses the geometric mean of the sample vector as the reference, a CoDA analysis framework formulated by Aitchison 1986. We followed the methods laid out in Quinn et al. 2019 and have described the detailed analysis in the Materials and Methods Line 171- 191.

We carried out the correlational analysis between environmental variables and populations of T6SS-bearing bacteria after clr transformations using Redundancy Analysis (RDA), a variant of PCA, as the data are now symmetrical and linearly related and amenable for correlational analysis. Details of the statistical methods were described under Materials and Methods Line 203-223.

*Given these inherent shortcomings of relative abundances as a metric, I am not convinced that the measured changes in relative abundances can be linked to an interaction between species here. If the analysis the authors did accounts for what is discussed in the paper above, I suggest this be addressed in this manuscript as this figure immediately raised a red flag in my mind.*

- The interactions derive from figure 6 were putative interactions, where hypothesis was formulated to guide actual, live experiments of the interaction between T6SS-bearing bacteria and other bacterial groups. Even though that is the intent of the graph, we agree that the nature of the data (based on sequencing and relative abundance) may not be robust to produce those putative hypotheses. We have removed Figure 6 in the revised manuscript in this regard, and even in doing so, it did not diminish the main message of the paper.

*The same point of criticism applies to relative abundances as used in Fig. 2 in this study, and I’d be interested to hear the authors’ point of view on how their analysis might be generally influenced by the intrinsic shortcomings of relative abundance data.*

As for Fig 2, we re-did the analysis by doing centered log ratio transformation and the transformed data was plotted as a heatmap to visualized how the OTUs have changed in abundance relative to the rest of the bacterial community (which is using geometric mean as a reference, based on compositional analysis by Aitchison 1986). By doing this, we only found a slight change in the result and interpretation of the data, and we have made this modification accordingly in the text. See Lines 260 – 275.

*b) Labelling bacterial genera containing T6SS-structural genes as “predatory” (throughout the manuscript). “Predation” in the strict sense implies not only killing, but also consumption of the targeted cells and nutritional exploitation (as the authors explain in the introduction). Although the T6SS is putatively involved in some bacterial predatory phenotypes sensu strictu (e.g. in Myxococcus), the T6SS is extremely wide-spread among Gram-negative bacteria and a role for it in actual “predation” has not been shown for any other species to the best of my knowledge. In the vast majority of species where the T6SS has been studied mechanistically in the lab in great detail, the T6SS is used as anti-competitor weaponry to kill competitors, where the main benefit is not nutrient uptake from the killed cell but rather just the physical removal of a competitor, which leads to more space for growth, and decreased nutrient-based competition. The chain of showing predation would need to include: find T6SS genes --> show that T6SS is expressed --> show that T6SS kills competitors --> show that nutrients from killed cells are taken up and significantly enhance fitness of the attacker. Since the study at hand only focusses on finding T6SS genes, I suggest the label “predatory” bacteria is removed and replaced by a more conservative label, e.g. “T6SS-harbouring bacteria” or something along those lines. Similarly, instead of saying “predating on competitors” I would say “T6SS-dependent killing or -inhibiting of competitors”.*

-We have removed the term predatory bacteria in the revised texts and used T6SS- bearing bacteria as suggested. We also used the term interbacterial competition to refer to the interaction.
 *In summary, I think this study contains interesting results on the distribution of T6SS-genes in marine environments but could use with some more discussion on how representative relative abundances really are for environmental communities, and I would also suggest not labelling bacterial genera harbouring T6SS genes as “predatory bacteria”, as several aspects of this definition are not fulfilled by this study system.*

- We followed this suggestion of the reviewer and emphasized that our study focuses on the T6SS-bearing heterotrophic bacteria living in the marine water column or the marine pelagic environment, which to our knowledge, has rarely been addressed in the literature.  *Minor comments:

  1. L82 -84: “Information is currently scarce …” We do have quite a bit of information on how the T6SS affects survival and success in host-associated communities though (e.g. Vibrio in squids* [*https://www.pnas.org/content/115/36/E8528*](https://www.pnas.org/content/115/36/E8528)*; Salmonella in guts* [*https://pubmed.ncbi.nlm.nih.gov/27503894/*](https://pubmed.ncbi.nlm.nih.gov/27503894/)*; and others), which should fall under an “ecological setting” as the authors have phrased it here. We do indeed have less information on non-host-associated environments like the ocean, and if this is the gap that the authors would like to emphasize than I suggest to rephrase this statement accordingly.*
- As stated above, we have revised the paper and emphasized that our study focuses on T6SS-bearing bacteria living in the pelagic environment. For specifics, see lines 16-21 in the abstract, Line 79-82 in the introduction and have subsequently discussed the results and implications throughout the Discussion section.

*2. L84 Do --> does (T6SS most often written as singular, the T6SS as a whole)*

- This sentence or question has been removed in the introduction. As the reviewer suggested, sequence data may not be able to address this type of question, hence the removal of the statement/question.

*3. L103 bacteria populations --> bacterial populations*

-This has been corrected. See Line 93.

*4. L103 bacteria-bacteria --> Interbacterial (just a suggestion)*

- We have removed the sentence containing this hyphenated word in Line 94 (previously L103) but we have used this suggested wording throughout the paper when we talk about bacteria-bacteria competition. For example, see Line 78, 80, 441 etc.

*5. L104 in --> for*

- This has been corrected. See Line 94.

*6. L111 for --> for the*

- This has been corrected. See Line 100.

*7. L236 bacteria --> bacterial*

- This has been corrected. See Line 256.

*8. Fig. 2: I’m a bit confused: why are there gaps in the plot at certain time points? is it because no DNA was isolated here, no T6SS-containing genera were present here, or no samples were taken (but then why plot)?*

- The reviewer is correct, there were no samples taken at these time points. These empty datapoints were removed. We subsequently changed the graph into a heatmap figure to better show the differences in the occurrence of the T6SS-bearing bacteria through time. The data was treated as compositional dataset and were subjected to centered log-ratio transformation, which take into account the abundance changes of T6SS-bearing bacteria using the geometric mean per sample as reference. See revised Fig. 2.

*9. Fig 2. Legend: “Error bars … across those samples” Sample size should be indicated in all figure legends for figures that contain errors bars.*

- As mentioned above, the figure has been changed into a heatmap and all replicates for each sample were included in the figure. We have also indicated the sample size (duplicate and triplicate) in the figure legends. Please see legend in Fig 2 Line 283-284.

*10. L296 Fig. 4 is cited, but I think this should be Fig. 3 (the one with the global map)*

- Thank you for pointing out the mistake. We have corrected this as Fig. 3.

*11. L300 Fig. 4 Legend, but I think this should be Fig. 3 Legend*

Please see response above. The legend now says Fig 3. See Line 329.

*12. L339 p-value: throughout the study, are p-values corrected for multiple testing? If yes, this should be mentioned in the method section.*

-We have revised the analysis and used Benjamini-Hochberg correction for multiple testing and used the resultant p-values. See Line 196 in Materials and method sections, where we mentioned the method.

*13. L349-352: add units for all variables/environmental factors*

- We have added the units in the text of Fig 4 legend as suggested by the reviewer (see Lines 381), as well as in other places where they occur, such as in the results section/legends.

*14. L337+L339 + L366 p-values are > 0.05, which is above the significance threshold of 0.05 used in this study (or at least above all the thresholds I saw explicitly mentioned, e.g. in legends for Fig. 5, Fig. 6, and in the methods section). P-value cut-offs are of course inherently arbitrary, but if different cut-offs were used for different analyses this should be mentioned somewhere (e.g. in methods section). If these values are indeed considered “non-significant”, the claims based on the respective comparisons should be amended in the main text.*

- As the author mentioned, p-value cut-offs are arbitrary. As for any statistical analysis, we use the p-value cut-off of 0.05 (after correcting for multiple testing), when indicating a specific environmental variable as having a significant contribution to the structure of T6SS-bearing populations. As mentioned above, we re-did the analysis after clr transformation of the dataset using Redundancy analysis, which gave a robust multivariate correlation between the environmental determinants and T6SS-bearing bacteria. See Lines 343-413.

*15. L373-375: add units for all variables/environmental factors*

- Units have been added to the text in Fig. 5 legend. See Lines 41 to 424. We have also added the units in the results section/legends where they occur.

*16. Fig. 6 Legend – since two different datasets are used in this study (SC wharf vs. TARA) it would be good to indicate here which dataset this specific interaction analysis is based on.*

- Figure 6 has been removed from the revised paper.

*17. Fig. 6 title – I suggest changing the figure title to “putative predation networks”, since “predation network” strongly suggests that pairwise interaction assays were conducted experimentally, with cultured isolates.*

- Figure 6 has been removed from the revised paper.
